# Warfarin Anticoagulant Therapy: A Southern Italy Pharmacogenetics-Based Dosing Model

**DOI:** 10.1371/journal.pone.0071505

**Published:** 2013-08-26

**Authors:** Cristina Mazzaccara, Valeria Conti, Rosario Liguori, Vittorio Simeon, Mario Toriello, Angelo Severini, Corrado Perricone, Alfonso Meccariello, Pasquale Meccariello, Dino Franco Vitale, Amelia Filippelli, Lucia Sacchetti

**Affiliations:** 1 CEINGE– Biotecnologie Avanzate s.c.ar.l., Napoli, Italy; 2 Dipartimento di Medicina Molecolare e Biotecnologie Mediche, Università di Napoli Federico II, Napoli, Italy; 3 Dipartimento di Medicina e Chirurgia, Università di Salerno, Salerno, Italy; 4 Laboratorio di Ricerca Pre-clinica e Traslazionale Oncologica, Centro di Riferimento Oncologico della Basilicata, Istituto di Ricovero e Cura a Carattere Scientifico, Rionero in Vulture (Pz), Italy; 5 A.O.R.N. Santobono Pausillipon, Napoli, Italy; 6 Dipartimento di Medicina Interna, Università of Napoli Federico II, Napoli, Italy; 7 Fondazione Salvatore Maugeri, IRCCS Istituto di Campoli Telese, Benevento, Italy; Tor Vergata University of Rome, Italy

## Abstract

**Background and Aim:**

Warfarin is the most frequently prescribed anticoagulant worldwide. However, warfarin therapy is associated with a high risk of bleeding and thromboembolic events because of a large interindividual dose-response variability. We investigated the effect of genetic and non genetic factors on warfarin dosage in a South Italian population in the attempt to setup an algorithm easily applicable in the clinical practice.

**Materials and Methods:**

A total of 266 patients from Southern Italy affected by cardiovascular diseases were enrolled and their clinical and anamnestic data recorded. All patients were genotyped for CYP2C9*2,*3, CYP4F2*3, VKORC1 -1639 G>A by the TaqMan assay and for variants VKORC1 1173 C>T and VKORC1 3730 G>A by denaturing high performance liquid chromatography and direct sequencing. The effect of genetic and not genetic factors on warfarin dose variability was tested by multiple linear regression analysis, and an algorithm based on our data was established and then validated by the Jackknife procedure.

**Results:**

Warfarin dose variability was influenced, in decreasing order, by VKORC1-1639 G>A (29.7%), CYP2C9*3 (11.8%), age (8.5%), CYP2C9*2 (3.5%), gender (2.0%) and lastly CYP4F2*3 (1.7%); VKORC1 1173 C>T and VKORC1 3730 G>A exerted a slight effect (<1% each). Taken together, these factors accounted for 58.4% of the warfarin dose variability in our population. Data obtained with our algorithm significantly correlated with those predicted by the two online algorithms: Warfarin dosing and Pharmgkb (p<0.001; R^2^ = 0.805 and p<0.001; R^2^ = 0.773, respectively).

**Conclusions:**

Our algorithm, which is based on six polymorphisms, age and gender, is user-friendly and its application in clinical practice could improve the personalized management of patients undergoing warfarin therapy.

## Introduction

Warfarin sodium [1] is the most frequently prescribed anticoagulant for the primary and secondary prevention of thromboembolic disorders worldwide [2]–[4]. Despite the advent of new oral antithrombotic agents such as dabigatran, rivaroxaban, apixaban, which have proven to be cost-effective compared with warfarin in some clinical conditions [5], [6], warfarin remains the mainstay of treatment for patients with mechanical heart valves and patients noncompliant to new therapies because in these populations their efficacy have not been explored [7].

Warfarin inhibits the Vitamin K Epoxide Reductase Complex 1 (VKORC1) thus reducing the activities of vitamin K-dependent clotting factors II, VII, IX and X and coagulation. S-warfarin, the most active of the two (R- and S-) isomers in the administered drug, is mainly metabolized by the cytochrome P450 2C9 isoenzyme (CYP2C9) [8].

Notwithstanding its wide use, warfarin has a narrow therapeutic range and a large interindividual variability in the dose needed (1–20 mg/day) to obtain an adequate anticoagulation effect [4]. The latter is generally measured by the prothrombin international normalised ratio (INR) and its range is 2.0–3.0 or higher in at-high risk patients [9]. Inappropriate INR levels may result in significant bleeding or stroke (INR levels greater or lower than the target range, respectively), particularly during the first weeks of therapy (induction phase) [9]–[14]. To date, most clinicians prescribe 3–10 mg/day for the first 2–5 days, then switch to a maintenance dose established based on frequent INR monitoring [2], [11], [14]. Warfarin-induced adverse effects account for over 10% of all adverse drug reactions leading to hospital admissions [15].

The large interindividual variation in warfarin dose requirement is attributable to clinical, demographic, environmental factors (age, gender, body mass index, daily vitamin K intake, concomitant diseases, interaction between drugs, and smoking), and to genetic factors, which account for 40–60% of the variability [16]–[18]. Among genetic factors, single nucleotide polymorphisms (SNPs) in the CYP2C9 (Gene Bank Accession Number AY702706; chr.10q24) and in VKORC1 (Gene Bank Accession Number AY587020; chr.16p11.2) genes were first described as major contributors to dose-response variability. Subjects bearing polymorphisms in one or both of these genes require lower or higher warfarin doses than subjects bearing the wild-type genes to obtain an adequate anticoagulant effect [1], [8], [16], [19]–[22]. More recently, patients bearing a SNP (rs2108622) in the CYP4F2 gene (Gene Bank Accession Number AF22194; chr.19p13.12), which is the vitamin K_1_ oxidase involved in vitamin K_1_ metabolism, were found to require a warfarin dose slightly higher than normal [23]–[25] or similar to normal [26], [27]. Moreover, a meta-analysis revealed a statistically significant association between rs2108622 and the interindividual warfarin dose variation [28], [29]. However, it was annotated (www.pharmgkb.org) as a Level 1B clinical association, namely “a variant-drug combination where the preponderance of evidence shows an association. The association must be replicated in more than one cohort with significant p-values, and, preferably with a strong effect size”).

In 2007 and in 2010, the US Food and Drug Administration, Center for Drug Evaluation and Research, suggested that CYP2C9 and VKORC1 -1639 G>A gene polymorphisms be typed before starting warfarin therapy [30], and issued specific guidelines in this sense [31]. This prompted several clinical trials to evaluate the use of pharmacogenetic tests before starting warfarin therapy. It also prompted the development of warfarin-dosing algorithms that include genetic and non-genetic factors [32]. Notably, predictive algorithms must be based on data representative of the target population, and they should be validated. To date, few studies have evaluated the global effect of genetic and non-genetic factors on warfarin dosage in Italian subjects [1], [33]–[36].

The aim of this study was to estimate, in a Southern Italy population of subjects affected by cardiovascular disorders undergoing warfarin therapy, the effect of the CYP2C9 (*2 and *3), CYP4F2*3, VKORC1 (-1639 G>A, 1173 C>T and 3730 G>A SNPs combined with clinical status, demographic and environmental factors on warfarin dosing.

## Results

The clinical, anamnestic and demographic features of our warfarin-treated patients are shown in **Table 1**.

**Table 1:** Clinical, anamnestic and demographic features of the warfarin-treated patients.

Age *(years)*	67.35±11.05
Gender male	55.2%
BMI (kg/m^2^)	26.90±4.24
Indications for warfarin therapy	
*Cardiac valve replacement*	43.9%
*Atrial fibrillation*	38.1%
*Dilatative cardiomyopathy*	8.5%
*Deep venous thrombosis*	6.5%
*Pulmonary embolism*	3.0%
Smoking	8.7%
Liver disease	15.5%
Dyslipidaemia	65.2%
Hypertension	62.5%
Drug assumption	56.8%
Only drugs that increase the warfarin effect	33.0%
Only drugs that decrease the warfarin effect	17.0%
Both types of drugs	6.8%
No drugs	43.2%
Warfarin dose assumed (mg/week)	28.73±13.22

Continuous variables are expressed as means ± standard deviation (SD) and categorical variables as percentages.

Our population did not differ in terms of gender (55.2% male). Similarly, there were no differences between men and women in terms of age, body mass index and the other parameters evaluated (data not shown).

Cardiac valve replacement and atrial fibrillation were the most frequent cardiovascular indications (43.9% and 38.1%, respectively). Most patient (43.2%) did not assume any drug in addition to warfarin.

**Figure 1:**
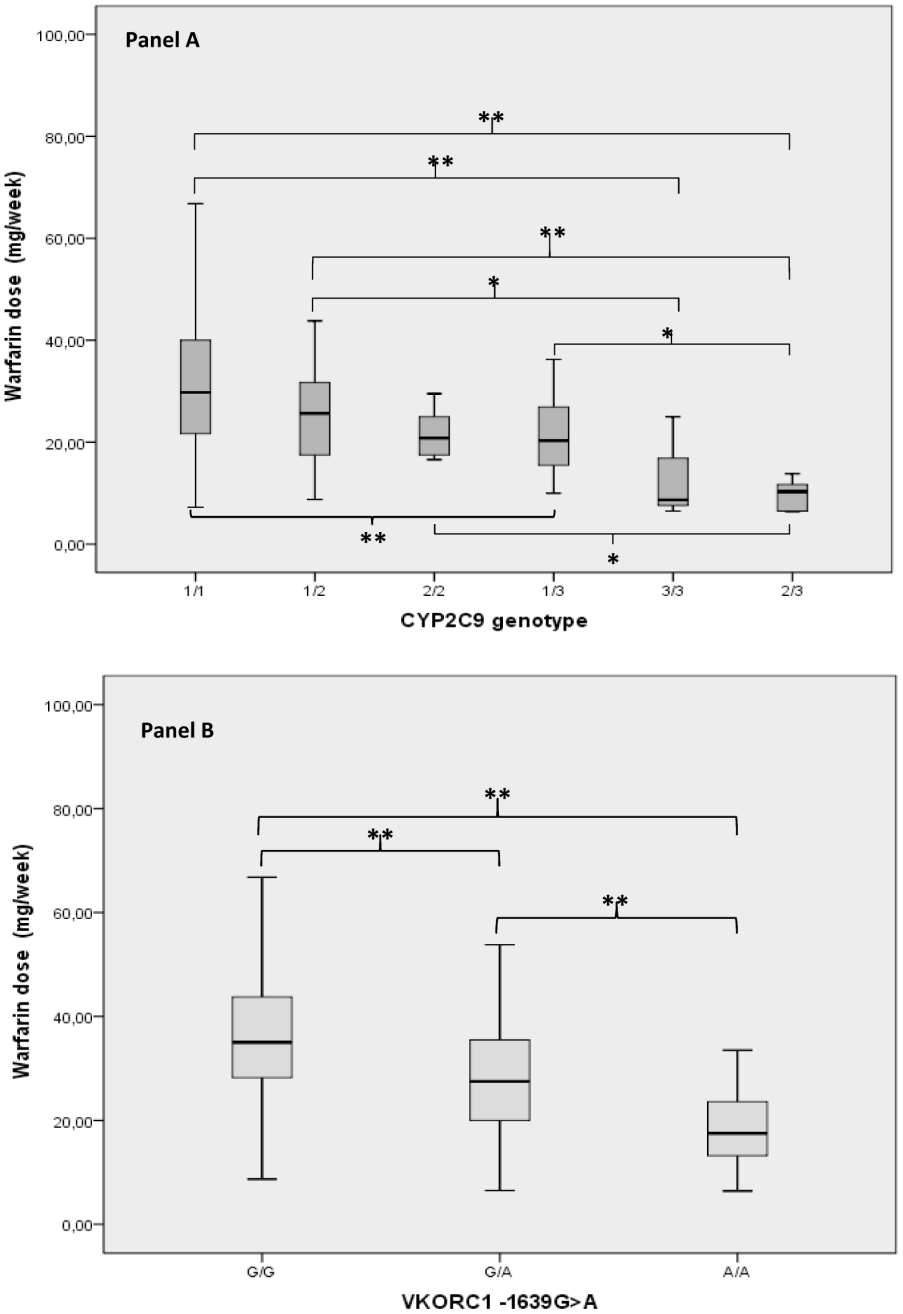
Relationship between the weekly warfarin dose and CYP2C9 (Panel A) or VKORC1 -1639G>A (Panel B) genotypes. Each box indicates the values from 25° to 75° percentile (interquartile range), the horizontal lines represent the median value of weekly warfarin dose, the maximum length of whisker is 1.5 fold the interquartile range. * p<0.05, ** p<0.001at ANOVA test.

Allelic and genotype frequencies of the CYP2C9*2, CYP2C9*3, CYP4F2*3, VKORC1-1639 G>A, VKORC1 1173 C>T and VKORC1 3730 G>A polymorphisms obtained in our patients and those reported in other Caucasian groups are reported in **Table 2**. Genotype frequencies, at the level of all tested genes, were in Hardy-Weinberg equilibrium. The comparison between the weekly warfarin dose assumed in the subjects bearing the wild-allele or the polymorphic CYP2C9 variants is shown in **Figure 1A**. Patients bearing the CYP2C9*1/*3, *2/*3 and *3/*3 genotypes required a significantly lower warfarin dose than patients with the wild-type allele (22.03 mg/week±8.80; 13.4 mg/week±10.10; 9.74 mg/week±3.25; respectively *vs* 32.11 mg/week±13.98; p<0.001).

**Table 2:** Allele and genotype frequencies of CYP2C9*2, CYP2C9*3, VKORC1 (-1639 G>A, 1173 C>T, 3730 G>A) and CYP4F2 1297G>A polymorphisms obtained in our population and in other Caucasian populations.

	Genotype frequencies			Allelic frequencies		
	Our data		Other studies*		Our data	Other studies*
*Gene*	*N*	*%*	*%(min-max)*		*%*	*%(min-max)*
**CYP2C9**						
* **1/** * **1**	159	60.2	56.4–66.9	*1	77.5	50.3–83.0
* **1/** * **2**	58	22.0	16.4–23.8	*2	15.7	11.9–32.0
* **1/** * **3**	33	12.5	8.9–12.7	*3	9.8	5.7 –17.2
* **2/** * **2**	6	2.3	1.7–2.3			
* **2/** * **3**	5	1.9	1.1–3.6			
* **3/** * **3**	3	1.1	0.3–9.1			
**CYP4F2 1297G>A**						
**G/G**	121	45.8	39.2–46.0	G	69.1	65.8–70.3
**G/A**	123	46.6	42.0–48.2	A	30.9	34.2–29.7
**A/A**	20	7.6	9.4–12.6			
**VKORC1 -1639G>A**						
**G/G**	67	25.4	32.2–37.3	G	50.2	58.2–59.4
**G/A**	131	49.6	46.9–55.1	A	49.8	40.6–41.8
**A/A**	66	25.0	7.6–20.8			
**VKORC1 1173C>T**						
**C/C**	114	43.2	26.4–40.8	C	65.1	57.8–62.2
**C/T**	116	43.9	43.2–50.8	T	34.9	37.8–42.2
**T/T**	34	12.9	8.3–25.0			
**VKORC1 3730G>A**						
**G/G**	132	50.0	38.2–48.0	G	71.4	62.6–66.4
**G/A**	113	42.8	39.5–52.7	A	28.6	37.4–33.6
**A/A**	19	7.2	4.0–15.0			

* Refs. [33]–[39].

**Figure 2:**
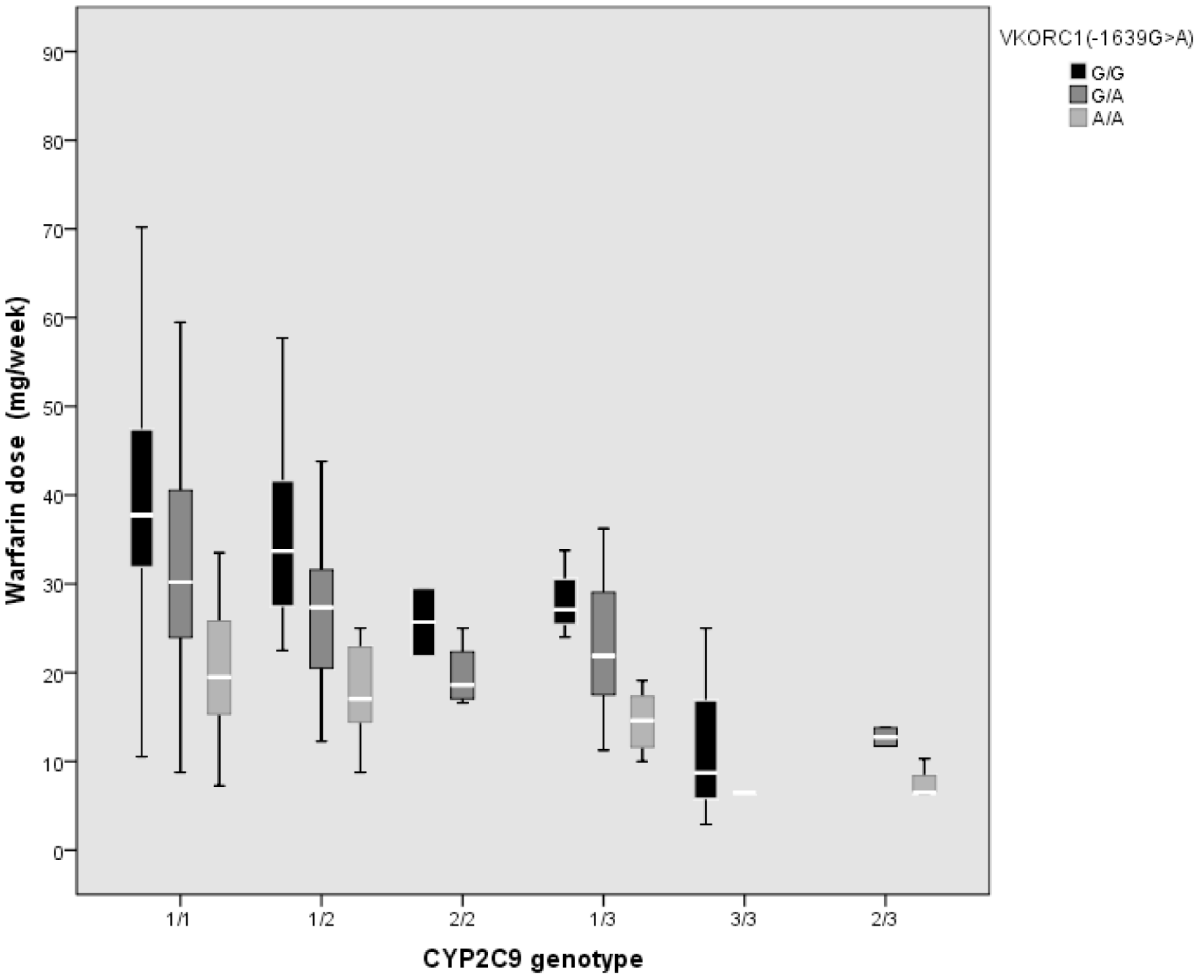
Combined effect of CYP2C9 and VKORC1 -1639 G>A polymorphic genotypes on stable weekly warfarin dose (mg/week). Each box indicates the values from 25° to 75° percentile (interquartile range), the white lines represent the median value of weekly warfarin dose, the maximum length of whisker is 1.5 fold the interquartile range. In detail below are shown the specific statistical significances for each comparison. CYP2C9 *1/*1+VKORC1 -1639 G/G *vs* CYP2C9 *1/*1+VKORC1 -1639A/A, p<0.001. CYP2C9 *1/*1+VKORC1 -1639 G/G *vs* CYP2C9 *1/*1+VKORC1 -1639G/A, p<0.05. CYP2C9 *1/*1+VKORC1 -1639 G/A *vs* CYP2C9 *1/*1+VKORC1 -1639A/A, p<0.001. CYP2C9 *1/*2+VKORC1 -1639 G/G *vs* CYP2C9 *1/*2+VKORC1 -1639A/A, p<0.001. CYP2C9 *1/*2+VKORC1 -1639 G/G *vs* CYP2C9 *1/*2+VKORC1 -1639G/A, p<0.05. CYP2C9 *1/*2+VKORC1 -1639 G/A *vs* CYP2C9 *1/*2+VKORC1 -1639A/A, p<0.001. CYP2C9 *1/*3+VKORC1 -1639 G/G *vs* CYP2C9 *1/*3+VKORC1 -1639A/A, p<0.05. CYP2C9 *1/*3+VKORC1 -1639 G/A *vs* CYP2C9 *1/*3+VKORC1 -1639A/A, p<0.05.

The mean weekly warfarin dose was also significantly lower in VKORC1 -1639 G>A mutated homozygotes and in heterozygotes than in patients with the wild-type allele (18.81 mg/week±7.98, 29.15 mg/week±11.79, and 37.80 mg/week±13.37, respectively, p<0.001) (**Figure 1B**). We also evaluated the additive effect of the CYP2C9 and VKORC1 -1639 G>A polymorphic genotypes on warfarin dose requirement. The simultaneous presence of these polymorphisms further significantly reduced the warfarin dose requirement as shown in **Figure 2**.

Slightly higher warfarin dosages were required by VKORC1 3730 G>A heterozygotes and homozygotes than by patients carrying the wild-type allele (32.56 mg/week±13.48 and 33.39 mg/week±16.08 *vs* 24.38 mg/week±11.12; p<0.05, respectively) (**Figure S1**). No difference in warfarin dosages was observed, by ANOVA analysis, in subjects bearing the VKORC1 1173 C>T (p = 0.72) or the CYP4F2*3 (p = 0.36) polymorphisms.

Haploview software showed the lack of linkage disequilibrium between VKORC1-1639 G>A and 1173 C>T (D′: 0.186). Using multiple linear regression analysis we assessed the effect of the genetic and non genetic factors (see Table 1) on warfarin dose, with the actual weekly warfarin dose as dependent variable. Using the Jackknife procedure, we then validated the algorithm developed on our data set. The percentage contributions of the various factors on warfarin dose were in decreasing order: 29.7% VKORC1-1639 G>A, 11.8% CYP2C9*3, 8.5% age, 3.5%, CYP2C9*2, 2.0% gender and 1.7% CYP4F2*3. The effects of VKORC1 1173 C>T and VKORC1 3730 G>A were marginal (<1% each). In our population, the above factors accounted for 58.4% of the variance in warfarin dosage (**Table 3**) and 57.2% after the exclusion of VKORC1 1173 C>T and VKORC1 3730 G>A, which were associated with the lowest and highest doses, respectively.

**Table 3:** Factors affecting weekly warfarin dose requirements in regression model*.

Variable	P value	Partial R^2^	Coefficient B (95% CI)
***VKORC1 -1639 G>A***		***29.7***	
-1639 G/A	0.439		−0.033 (−0.116, −0.050)
-1639 A/A	<0.0001		−0.278 (−0.335, −0.221)
***CYP2C9*** * ***3***		***11.8***	
(*1/*3)	<0.0001		−0.149 (−0.201, −0.097)
(*3/*3)	<0.0001		−0.475 (−0.672, −0.278)
***AGE***	<0.0001	***8.5***	−0.050 (−0.007, −0.003)
***CYP2C9*** * ***2***		***3.5***	
(*1/*2)	<0.0001		−0.090 (−0.132, −0.047)
(*2/*2)	0.009		−0.218 (−0.381, −0.054)
***GENDER***	0.001	***2.0***	0.061 (0.024,0.098)
***CYP4F2*** * ***3***		***1.7***	
(*1/*3)	0.121		0.030 (0.008,0.069)
(*3/*3)	0.015		0.087 (0.017,0.158)
***VKORC1 1173 C>T***		***0.8***	
1173 C/T	0.037		−0.085 (−0.165, −0.050)
1173 T/T	0.437		−0.022 (−0.079,0.034)
***VKORC1 3730 G>A***		***0.4***	
3730 G/A	0.022		0.054 (0.008,0.100)
3730 A/A	0.100		0.060 (−0.012,0.132)

* (Total R^2^ for the model 58.4%).

**Figure 3:**
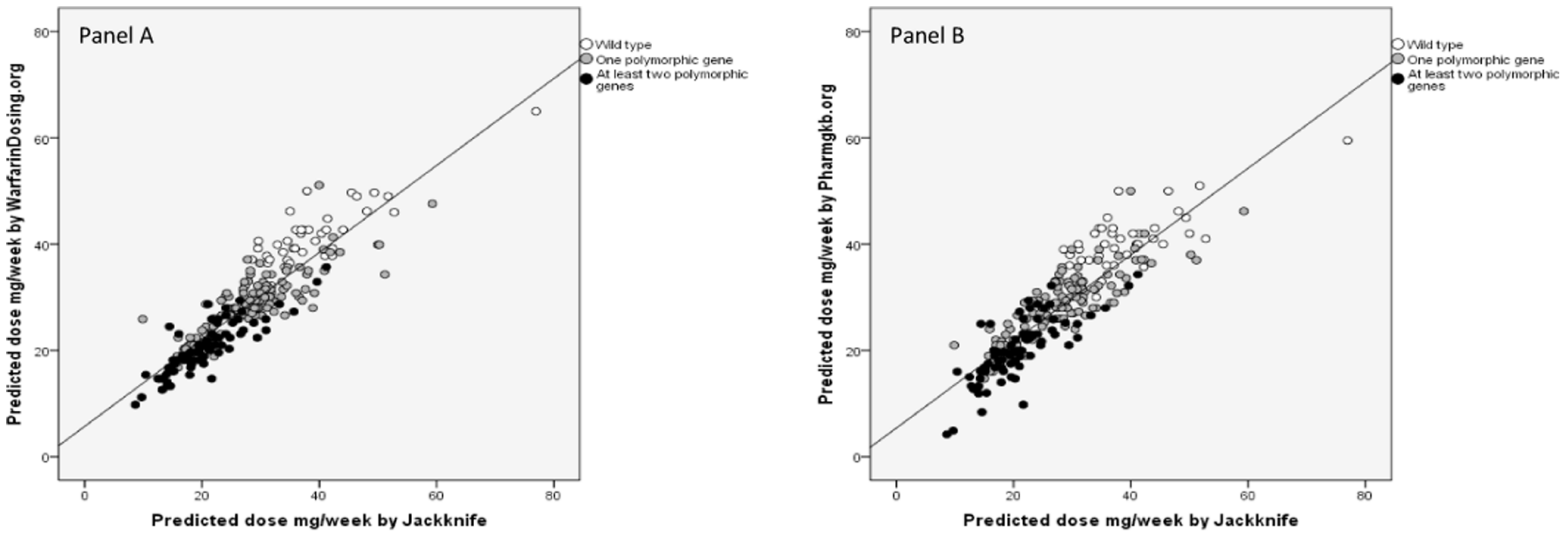
Correlation analysis (Pearson coefficient) between our predicted warfarin dosage by Jackknife and that predicted by www.warfarindosing.org (panel A, p<0.001, R^2^ = 0.805) and www.pharmgkb.org (panel B, p<0.001, R^2^ = 0.773). The solid line represent a good correlation between the two doses. Open circles represent individuals for whom no variants in CYP2C9 and VKORC1 -1639 G>A were detected. Gray circles represent individuals with only one polymorphic gene (either VKORC1 -1639 G>A or CYP2C9* 2 or *3). Black circles represent individuals with at least two polymorphic genes.

To explore how our algorithm worked versus the two online algorithms (www.warfarindosing.org and www.pharmgkb.org) (accessed September 2011), we compared by Pearson analysis each patient's predicted warfarin dosage by the Jackknife procedure with those predicted by the two online algorithms (**Figure 3A and 3B**). The data obtained with our algorithm significantly correlated with those predicted by two online algorithms: Warfarin dosing (p<0.001; R^2^ = 0.805) and Pharmgkb (p<0.001; R^2^ = 0.773).

## Discussion

We investigated the effect of genetic and not genetic factors on the mean weekly warfarin dose variability in an adult South Italian population to setup a simple algorithm easily applicable in clinical practice.

The allele and genotype frequencies of the CYP2C9*2, CYP2C9*3, CYP4F2*3 and VKORC1 (-1639 G>A, 3730 G>A and 1173 C>T) genes were similar to those found in other Caucasian populations, except for a slightly higher prevalence of the VKORC1 -1639 A/A genotype (25% vs 7.6–20.8%) [33]–[39].

The effect of the CYP2C9, CYP4F2 and VKORC1 genotypes on warfarin dose was similar to those previously reported [10], [12], [40]. In particular, the warfarin dose was 17.0% and 32.0% respectively lower in subjects bearing the CYP2C9*2 or CYP2C9*3 polymorphic alleles versus wild type, which is similar to the previously reported reductions of 18–20% and 34–38%, respectively [10], [38], [40]–[42] in Caucasians. Furthermore, there was a 25% dose reduction in subjects bearing the VKORC1-1639 A allele, which is also in agreement with previously reported percentages (25–30%) [12]. The mean weekly warfarin dose was also lower in our patients bearing both the CYP2C9 and VKORC1-1639 G>A polymorphic genotypes, namely between 34.8% and 84.0% lower than in wild-type patients, which compares well with previously reported reductions (34%–75% and 41%–79%) [2], [30]. In the two previous studies that typed smaller than our Italian populations (148/147 vs 266 patients) [1], [35] for VKORC1 1173 C>T but not for -1639 G>A SNP, a different degree of association between VKORC1 1173C>T and warfarin dosing was observed, 0.8% (this study) vs 20% and 13,8% [1], [35]. These data support a large population-based variability in gene polymorphism-dependent warfarin dosing.

In our population, we did not find any linkage disequilibrium (D′: 0.186) between VKORC1-1639 G>A and 1173 C>T, in contrast with those reported in other ethnic groups [43], [44]. Population differences in minor allele frequencies observed at level of the tested polymorphisms VKORC1 -1639G>A and 1173 C>T could drive interethnic differences detected among Caucasian populations, also from different Italian regions [1], [35], and these genetic factors together to different cultural and lifestyle factors could in part explain the above discrepancies. Higher warfarin doses were required by both the heterozygous and mutated homozygous VKORC1 3730 G>A patients with respect to subjects carrying the wild-type allele, which suggests that this polymorphism has less impact on warfarin dosage than VKORC1-1639 G>A, in agreement with the meta-analysis reported by Yang et al. [38].

In the regression model, the variant CYP4F2*3 polymorphism entered with an R^2^ of 1.7%, and the difference in warfarin dose between CYP4F2 A/A *vs* CYP4F2 wild type was 0.6 mg/day. This observation is in line with a previous finding that CYP4F2*3 has only a small effect on warfarin dose variability [23]. However, the effect of CYP4F2*3 on warfarin dose-response variability is debatable; in fact, it ranges from 1%–7% [23], [33], [34] to not significant [26], [27].

Among non genetic factors, regression analysis revealed that age (p<0.0001; R^2^ = 8.5%) and gender (p = 0.001; R^2^ = 2.0%) contributed to the overall variability in warfarin dose, which is in agreement with a previous report [34]. Warfarin dosages predicted by our algorithm significantly correlated with those predicted by the Warfarindosing and Pharmgkb algorithms. The algorithms explain similar to ours the percentages of the warfarin response (47%–58%) in other Caucasian populations [45]–[46], but they use more data.

In conclusion, by exploring the most relevant genetic variants and by applying a user-friendly algorithm, our study contributes to the field of warfarin pharmacogenetics in a Southern Italy population. One may envisage that a genotype-guided and clinical-guided (versus clinical-guided) warfarin dosing algorithm could improve patient care in terms of dosage particularly in the initial phase of therapy, resulting in a decreased time below the therapeutic range and consequently in a reduction of adverse drug reactions.

## Materials and Methods

### Subjects

Two hundred and sixty-six warfarin-treated patients from Southern Italy, 45% female, were enrolled at the Department of Internal Medicine, University of Naples Federico II, at the Foundation Salvatore Maugeri IRCCS Istitute of Campoli Telese, Benevento, at the Santobono Pausilipon Hospital, Naples, and at the Department of Experimental Medicine, Second University of Naples, Italy. The study was performed according to the second Helsinki Declaration, all subjects provided written informed consent to participate in the study which was approved by the Ethics Committees of the above institutions. At enrolment all patients had been taking a stable dose of warfarin for at least 3 months, which is warfarin dose to achieve INR 2–3. Anamnestic, clinical and lifestyle information were recorded on a structured interview form. Hypertension, systolic blood pressure above 130 mmHg and diastolic blood pressure above 85 mmHg, and body mass index (body weight [kg] divided by squared height [m^2^]) were also recorded. Liver dysfunction (aspartate aminotransferase >35 U/L women, >40 U/L men; alanine aminotransferase >35 U/L women, >40 U/L men), and dyslipidemia (serum total cholesterol and/or triglycerides levels above 190 mg/dL or 150 mg/dL, respectively) were also measured.

### Samples and Methods

Three fasted blood samples (one with EDTA for DNA extraction, one with sodium citrate and one without anticoagulant for haematological and biochemical investigation, respectively) were collected from each patient. DNA was extracted with the Nucleon BACC2 kit (Amersham Life Science, England). Coagulation and biochemical tests were performed by routinely methods using reagent and equipment from Siemens, (Germany) and from Roche Diagnostics (Germany), respectively.

We genotyped patients for the CYP2C9 (CYP2C9*2, rs1799853, exon 3, c.430 C>T, p.Arg144Cys; CYP2C9*3, rs1057910, exon 7, c.1075 A>C, p.Ile359Leu), CYP4F2*3 (rs2108622, c.1297G>A, p.V433M) and VKORC1 -1639 G>A (rs9923231) (also known as 3673 G>A) polymorphisms, together with positive and negative quality control samples, using the Real-Time TaqMan method [47], [48] and commercial kits, namely Pre-developed TaqMan Assay Reagents Human Allelic Discrimination (CYP2C9*2 and *3) (probe code 4312568 and 4312569) and TaqMan Drug Metabolism Genotyping Assay (CYP4F2*3 and VKORC1) Applied Biosystems, CA, USA.

The PCR was set up in a 96-well plate with a 25 µL mix reaction, 10–20 ng of genomic DNA per assay. The amplification protocol was performed according to the manufacturer's indications. Variants VKORC1 1173 C>T, (rs9934438) (also known as 6484 C>T) and VKORC1 3730 G>A, (rs7294) (also known as 9041G>A) were detected by denaturing high performance liquid chromatography on Wave 2.0 Transgenomic instruments (Omaha, NE, USA). Each suspicious chromatogram was then sequenced. The PCR primers and conditions are listed in **Appendix S1**.

### Statistical Analysis

The Hardy-Weinberg equilibrium was verified for all investigated polymorphisms by the χ^2^ test.

The Kolmogorov-Smirnov test was performed to evaluate the distribution of continuous variables.

Data were expressed as average ± standard deviation (SD) (continuous variables) or in percentage (categorical variables). We evaluated differences of clinical and genetic variables among groups by the Student *t* test and analysis of variance (ANOVA), followed by post hoc test with Bonferroni correction. A *p*<0.05 was considered statistically significant. Linkage analysis was performed by Haploview 4.0 software [49]. Multivariate linear regression was performed to identify the factors associated with the weekly warfarin dose expressed on a logarithmic scale. Global and partial R2 were measured, these latters assessing the percentage of the dose variability explained by the full model and by each factor included in the model. In order to obtain an unbiased estimate of the prediction ability of our algorithm we validated it using the Jackknife procedure [50], i.e., the predicted dose of each patient was obtained using the linear coefficients developed using the remaining patients in the data set, thus avoiding the bias introduced by scoring a patient with coefficients optimized with data of the patient himself. Weekly warfarin dose predictions were also obtained by two dosing algorithms published by the Warfarin Dose Refinement Collaboration (www.warfarindosing.org, accessed September 2011) and by the International Warfarin Pharmacogenetics Consortium (www.pharmgkb.org, accessed September 2011). These predicted doses were then correlated with those obtained by our validated algorithm. Statistical analysis was performed with the STATA 11.2 software (StataCorp LP).

## Supporting Information

Appendix S1 Specific PCR primers (for: VKORC1 1173 C>T and VKORC1 3730G>A) and conditions.
(DOC)

Figure S1 Relationship between the weekly warfarin dose and VKORC1 3730 G>A genotypes. Each box indicates the values from 25° to 75° percentile (interquartile range), the black central line represents the median value of weekly warfarin dose, the maximum length of whisker is 1.5 fold the interquartile range. * p<0.05.
(PDF)
