# Supplementary material for: Warfarin Anticoagulant Therapy: A Southern Italy Pharmacogenetics-Based Dosing Model
Source: PLoS One. 2013 Aug 26;8(8):e71505. doi: 10.1371/journal.pone.0071505 (PMC3753327; doi:10.1371/journal.pone.0071505)
Supplement: Appendix S1 — Specific PCR primers (for: VKORC1 1173 C>T and VKORC1 3730G>A) and conditions. (DOC) [file pone.0071505.s001.doc]

**Appendix S1: Specific PCR primers (for: VKORC1 1173 C>T and VKORC1 3730G>A) and conditions**

VKORC1 1173 C>T forward: 5’TGACATGGAATCCTGACGTG3’

VKORC1 1173 C>T reverse: 5’TAGTGTGTAGAAGATGCAACC3’

VKORC1 3730 G>A forward: 5’GCTTTGCTTTGGCATGTGAG3’

VKORC1 3730 G>A reverse: 5’ACACATGGTTCAGACTTGGC3’

PCR was performed using a final volume of 30 µl, containing approximately 50 ng of genomic DNA in 0.2 ml tubes (Eppendorf, Hamburg, Germany). The reaction mixture containing 67 mM Tris-HCl, 16.6 mM (NH_4_)_2_SO_4_, 5 mM MgCl2, 0.01%, 200 µM each of the four deoxyribonucleotides, 0.4 µM of oligonucleotide primers (see the sequences above) and 1U of eurotaq (Euroclone, Milan, Italy). Thermal cycling was performed in an Cycler TM Thermal Cycler (Bio-Rad, Richmond, CA) at the following temperature-time profile: 95°C for 5 min, followed by 35 cycles of 94°C for 30 seconds, 60°C for 90 seconds, and 72°C for 30 seconds. A final extension step of 5 min at 72°C completed the reaction.
