# Supplementary figures and images for: Warfarin Anticoagulant Therapy: A Southern Italy Pharmacogenetics-Based Dosing Model
Source: PLoS One. 2013 Aug 26;8(8):e71505. doi: 10.1371/journal.pone.0071505 (PMC3753327; doi:10.1371/journal.pone.0071505)

**Figure S1**

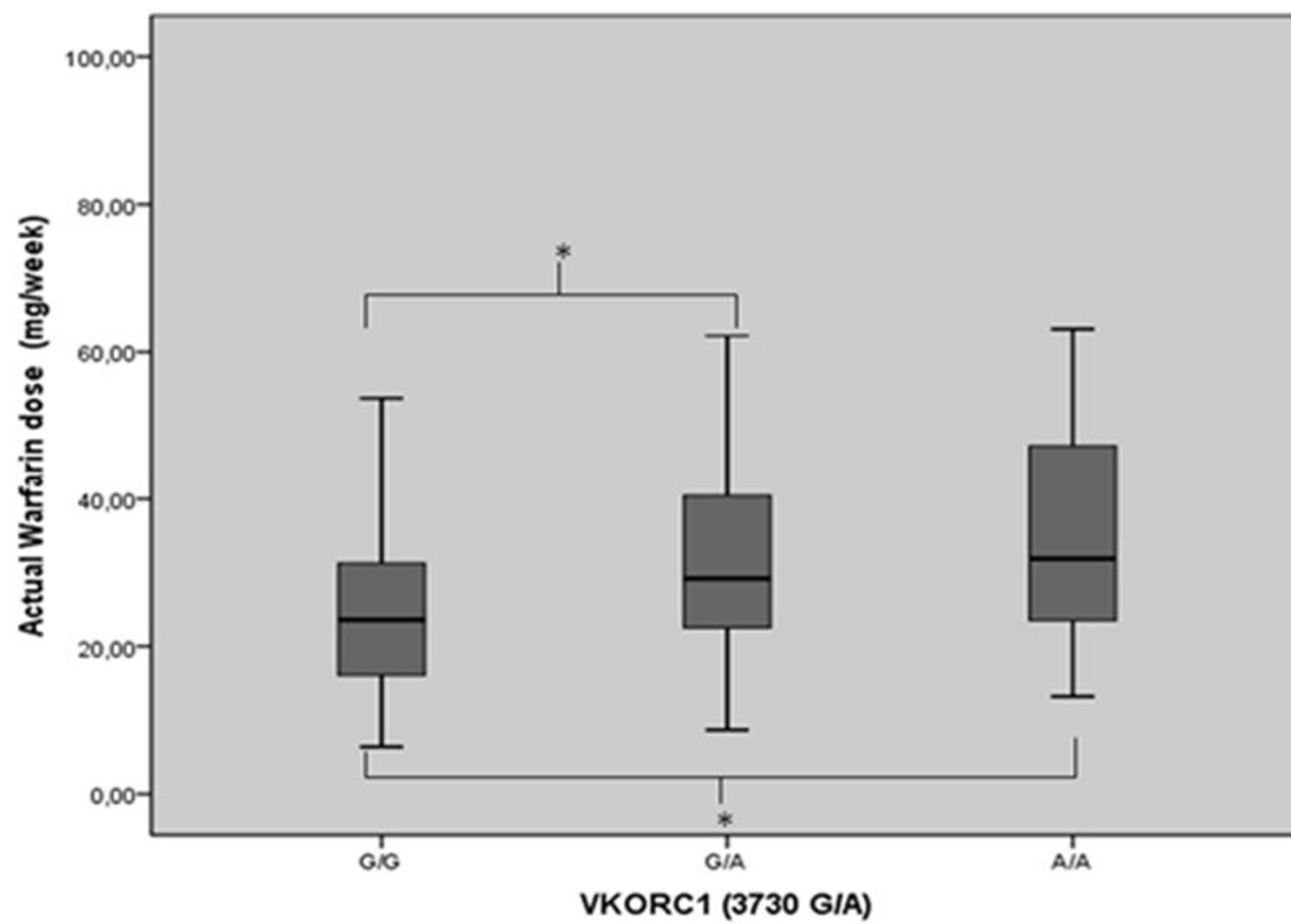

Supplement: Figure S1 — Relationship between the weekly warfarin dose and VKORC1 3730 G>A genotypes. Each box indicates the values from 25° to 75° percentile (interquartile range), the black central line represents the median value of weekly warfarin dose, the maximum length of whisker is 1.5 fold the interquartile range. * p<0.05. (PDF) [file pone.0071505.s002.pdf]
